# Leiomyoma of the bladder presenting as acute urinary retention in a female patient: urodynamic analysis of lower urinary tract symptom; a case report

**DOI:** 10.1186/1471-2490-10-13

**Published:** 2010-08-04

**Authors:** Masashi Matsushima, Hirotaka Asakura, Hirofumi Sakamoto, Minoru Horinaga, Yoko Nakahira, Hitoshi Yanaihara

**Affiliations:** 1 Department of Urology, Saitama Medical School, Moroyama, Saitama, Japan; 2 Department of Urology, Keio University School of Medicine, Tokyo, Japan

## Abstract

**Background:**

Most bladder tumors are derived from the urothelium. Benign mesenchymal tumors are rare. Leiomyoma of the bladder is the most common benign neoplasm. We present a case of leiomyoma of the bladder presenting with acute urinary retention in a female patient and report on the post-operative change in urodynamic findings. To our knowledge, few cases of this kind have been reported.

**Case Presentation:**

A 56-year-old woman presented with acute urinary retention. Evaluations including ultrasound, magnetic resonance imaging, cystoscopy, and urodynamics contributed to a diagnosis of leiomyoma of the bladder. Various medications were ineffective for solving her lower urinary tract symptoms; therefore, a transurethral resection was performed. The final pathological report was leiomyoma. After the operation, her symptoms resolved; this improvement was confirmed by an urodynamic analysis. The postoperative urodynamics demonstrated a lower frequency of detrusor overactivity during filling cystometry and an increase in the uroflow rate, with reduced detrusor pressure in a pressure flow study.

**Conclusions:**

Leiomyoma of the bladder can cause female outlet obstruction. A review of the literature and disease management is discussed.

## Background

Most bladder tumors are derived from the urothelium. Benign mesenchymal tumors are rare and comprise 1 to 5% of all bladder neoplasms [1]. Among them, leiomyoma is the most common benign neoplasm, accounting for 0.43% of bladder tumors [2]. Approximately 75% of the patients are young or middle aged [3]. We present a case of leiomyoma of the bladder with acute urinary retention in a female patient and report on the urodynamic changes after transurethral resection (TUR). A literature search of PubMed using the terms leiomyoma of the bladder and urodynamics suggested that only one other case of a bladder leiomyoma with acute urinary retention in a female patient who was evaluated using urodynamics has been previously published [4]. To our knowledge, there are few cases describing an urodynamic evaluation of a female patient with a bladder leiomyoma. Here, we present and discuss an exemplary case of lower urinary tract symptom (LUTS) caused by a leiomyoma of the bladder.

### Case Presentation

**Figure 1:**
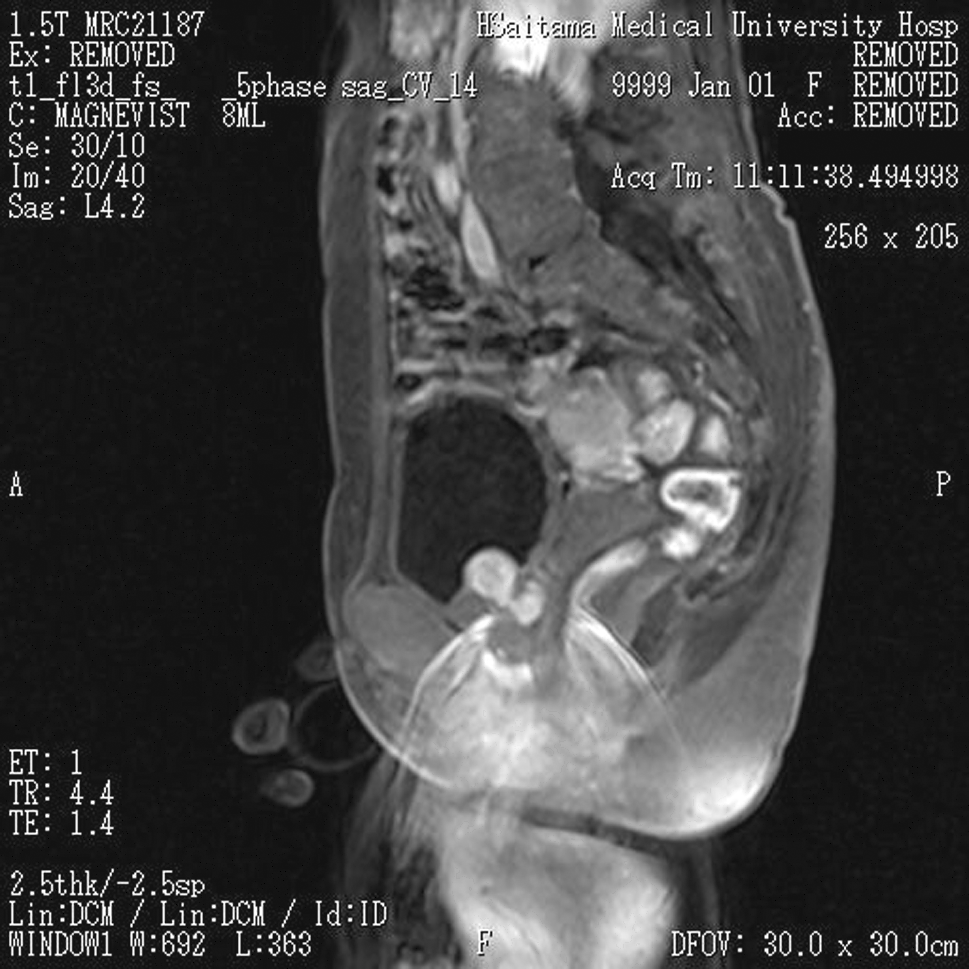
**Sagittal section of a magnetic resonance image**. Sagittal section of a magnetic resonance image shows a homogeneous mass (2.7 cm in diameter) occupying the area between the bladder neck and the anterior vaginal wall. The tumor mass was homogenously enhanced after the injection of gadolinium.

A 56-year-old woman presented at our hospital with an episode of acute urinary retention. She also complained of urinary frequency and urgency for the past 4 months. A physical examination did not reveal any particular findings, including pelvic organ prolapse. The results of a laboratory evaluation were within the normal limits. The urinary cytology was class II. In our cytology system, a scale of 1 - 5 was used. An ultrasonography detected a round mass in the bladder and no signs of hydronephrosis. A cystoscopy revealed a smooth surface and an intact mucosa tumor at the bladder neck. Magnetic resonance imaging (MRI) demonstrated a homogenous mass measuring 2.7 cm in diameter occupying the space between the bladder neck and the anterior vaginal wall. The tumor was homogenously enhanced after the injection of gadolinium (Figure. 1).

From these finding, we suspected a leiomyoma of the bladder. To confirm this diagnosis, we performed a transvaginal needle biopsy under ultrasonic guidance; however, the histological studies were unable to provide a diagnosis because of inadequate tissue collection during the biopsy.

The patient was treated with an anti-cholinergic agent and an alpha-blocker for 2 months, but her LUTS symptoms did not resolve. Therefore, we performed a TUR to resolve her symptoms and to confirm the pathological diagnosis. The TUR was successfully completed, and the patient's post-operative recovery was uneventful. Her LUTS were resolved a week after the operation.

**Figure 2:**
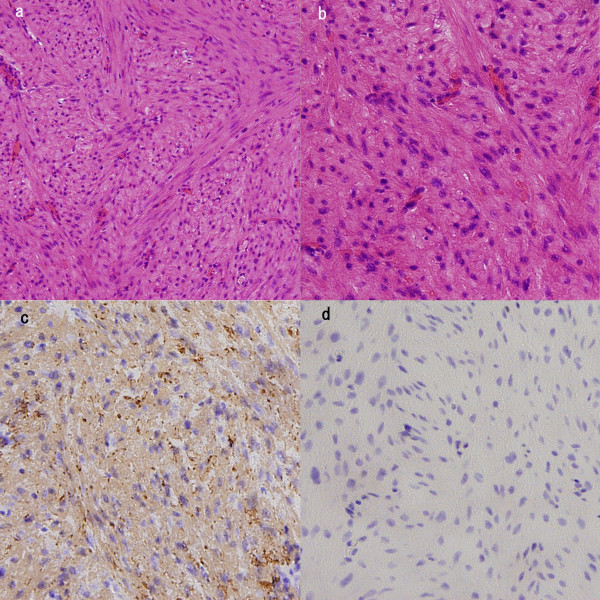
**Histopathological examination**. a, b) Histopathological examination of the tumor specimen shows a proliferation of spindle-shaped cells with eosinophilic cytoplasm and fibers (H&E). No evidence of mitotic figures or atypia was seen. c, d) Immunohistochemistry revealed positive staining for smooth muscle actin (c) and negative staining for Ki-67 (d). The tumor was diagnosed as a leiomyoma.

The pathological findings revealed the proliferation of spindle-shaped cells with eosinophilic cytoplasm and muscular and fibrous tissue with fibrous stroma. The nuclei of the cells were cigar-shaped and centrally located. No evidence of mitotic figures, coagulative T-cell necrosis or atypia was seen. Immunohistochemistry showed a positive expression for smooth muscle actin and a negative expression for Ki-67. These findings were consistent with a diagnosis of benign leiomyoma (Figure. 2). The final diagnosis was leiomyoma of the bladder without a malignant component.

**Figure 3:**
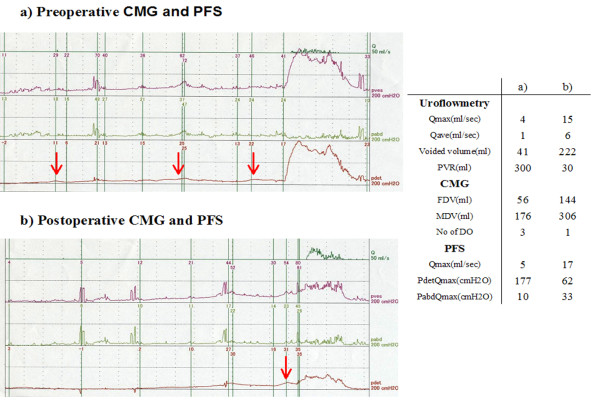
**Cystometry and pressure-flow study (pre-TUR and post-TUR)**. a) Preoperative-UDS: Three involuntary detrusor contractions occurred at 42, 138, and 160 mL during filling. She voided with a Q_max _of 4 mL/sec and a P_det _of 177 cmH_2_O. These values suggested female BOO. b) Postoperative UDS: Only one involuntary detrusor contraction occurred at 286 mL, just before voiding. Her maximum desire-to-void volume increased to 306 mL. She voided with a Q_max _of 15 cm/s and a P_det _of 62 cmH_2_O. A postoperative PFS demonstrated a considerable improvement in voiding. Q: flow, P_ves_: vesical pressure, P_abd_: abdominal pressure, P_det_: detrusor pressure, red arrows: voluntary bladder contraction.

We performed pre and post-operative (after three months) urodynamic studies (UDS) in this patient. The number of involuntary detrusor contractions decreased from 3 to 1 during a filling cystometry, and the values of first desire to void volume and maximum desire to void volume increased. Preoperatively, she voided with a Q_max _of 4 mL/sec and a P_det _of 177 cmH_2_O. Postoperatively, she voided with a Q_max _of 15 cm/s and a P_det _of 62 cmH_2_O. The preoperative pressure flow study (PFS) indicated female bladder outlet obstruction (BOO), but the post-operative PFS did not (Figure. 3). The urodynamic criteria for female BOO in this study were a pressure flow cutoff value of 15 mL/s or less and a P_det_Q_max _of 20 cmH_2_O or greater [5].

## Discussion

Mesenchymal tumors of the bladder, especially leiomyomas, are a relatively rare and heterogenous group of neoplasms arising from the mesenchymal tissues normally found in the bladder and constitute 1 to 5% of all bladder neoplasms [1]. Leiomyomas account for < 0.43% of all bladder tumors [2].

About 250 cases of leiomyoma of the bladder have been previously reported in the English language scientific literature [6]. The incidence of leiomyoma of the bladder is approximately three times higher in women than in men [7].

Leiomyoma of the bladder can be totally asymptomatic or can present in a varied manner depending on the location of the tumor with obstructive symptoms (49%), irritative symptoms (38%), hematuria (11%), and flank pain (13%) [7]. Nineteen percent of women with leiomyoma of the bladder are asymptomatic. Only few cases of leiomyoma of the bladder with acute urinary retention in a female patient have been previously reported.

Ultrasonography, MRI and cystoscopy are valuable diagnostic tools that can depict the morphology and anatomic location of leiomyomas. MRI is especially useful. Non-degenerative leiomyomas are usually visualized on MRI as low-intensity masses both on T1 and T2 weighted sequences with a smooth surface, while degenerative leiomyomas have a heterogenous signal intensity [8]. A variable pattern of enhancement is observed after the injection of gadolinium: some leiomyomas are homogenously enhanced, while other are not [3,9]. In the present case, MRI demonstrated a homogenous mass and clearly delineated the relationship of the mass to the bladder neck and anterior vaginal wall. However, MRI cannot completely differentiate leiomyomas from their malignant counterparts, leiomyosarcomas, especially when the lesion is degenerated or ulcerated. Thus, histological confirmation, such as a TUR, is necessary. In the present case, we performed an echo-guided transvaginal biopsy under local anesthesia on an outpatient basis. However, the biopsy failed because we could not obtain a sufficient amount of biopsy material. Perhaps we should have performed a TUR-biopsy instead of the needle biopsy.

Generally, the treatment for leiomyoma of the bladder involves a simple excision of the tumor. These lesions are sometimes resected transurethrally or transvaginally, but open surgery (tumor enucleation or partial cystectomy or total cystectomy) has also been reported. There are only a few reported recurrences and none of malignant degeneration [6]. therefore, the removal of the tumor itself is regarded as a sufficient treatment. Recently, an increasing number of cases in which TUR was performed have been reported.

Recent literature on BOO in women suggest that this condition might be more common than previously thought [10]. Leiomyomas of the bladder can compress the urethra, resulting in voiding dysfunction. However, few reports concerning the effects of surgical intervention on urodynamic changes in women with bladder leiomyomas have been made so far [4]. In the present case, a postoperative UDS demonstrated that the patient did not have a BOO and that she developed detrusor overactivity less frequently, confirming that the TUR procedure resolved her voiding and storage symptoms. Though her postoperative maximal flow rate is in the normal range, her P_det _is still relatively high. The high P_det_Q_max _after TUR is difficult to explain. At our institute, the normal P_det_Q_max _(average ± SD) value for healthy women is 26 ± 8 cmH2O. We generally perform a PFS twice and compare the non-tubed uroflowmetry results for each patient. Therefore, this high P_det_Q_max _value was reproducible. We speculate that the existence of an anatomical or functional urethral stricture may explain the high P_det_Q_max _value. We did not feel any resistance during the insertion of a 22-Fr cystoscope; therefore, an anatomical urethral stricture is unlikely. Thus, a functional urethral stricture might have caused the high P_det_Q_max _value, although we did not perform a video-urodynamics study to confirm the presence of a functional urethral stricture.

## Conclusions

Leiomyoma of the bladder can cause female outlet obstruction. Preoperative urodynamic assessments of this condition should permit a greater understanding of this unusual clinical entity.

## Consent

Written informed consent was obtained from the patient for publication of this case report and any accompanying images. A copy of the written consent is available for review by the Editor-in-Chief of this journal.

## List of abbreviations used

TUR: transurethral resection; LUTS: lower urinary tract symptom; MRI: Magnetic resonance imaging; UDS: urodynamic studies; PFS: pressure flow study; BOO: bladder outlet obstruction

## Competing interests

The authors declare that they have no competing interests.

## Authors' contributions

MM drafted the first manuscript. MM, HA and HS cared for the patient. HY helped to draft the manuscript. All authors reviewed the report and approved the final version of the manuscript.

## Pre-publication history

The pre-publication history for this paper can be accessed here:

http://www.biomedcentral.com/1471-2490/10/13/prepub
